# Editorial Notes

**Published:** 1929

**Authors:** 


### Editorial Note.

---

**William Harvey and Kingsweston.**

At the January meeting of the Bath and Bristol Branch of the British Medical Association a kinematograph film was exhibited showing the experiments which Harvey used to prove his theory of the circulation of the blood. This film was prepared by Sir Thomas Lewis and Dr. H. H. Dale for the celebrations held last year by the Royal College of Physicians to commemorate the tercentenary of the publication of William Harvey's *Exercitatio anatomica de motu cordis et sanguinis in animalibus.* It brought home convincingly the simplicity of the demonstrations which Harvey's great genius had conceived.

It is interesting to recall that at Kingsweston House near Bristol there is a contemporary portrait of Harvey, a replica apparently of one which hangs in the Kent and Canterbury Hospital. The picture is one of a group of family portraits placed in the great hall of Kingsweston by Edward Southwell, who died in 1755.

Kingsweston, which is now the property of Dr. Napier Miles, was formerly the seat of the Southwell family.

Sir Robert Southwell (1642–1702) married Elizabeth Dering, grand-daughter of William Harvey's brother, Daniel: so that it was his great-grand-uncle's portrait that Edward Southwell placed in the panelling at Kingsweston.

Sir Robert Southwell was a Privy Councillor, and had been Secretary of State for Ireland, and three times President of the Royal Society. He had studied medicine at Oxford and anatomy abroad.

The Rev. Richard Warner, in his *Excursions from Bath*, 1801, attributed the picture of Harvey to Sir Peter Lely. The Canterbury portrait has been attributed by Dr. F. William Cock to Cornelius Janssen.

